# The impact of urinary stone disease and their treatment on patients’ quality of life: a qualitative study

**DOI:** 10.1007/s00240-019-01142-0

**Published:** 2019-06-25

**Authors:** Aditya Raja, Fiona Wood, Hrishi B. Joshi

**Affiliations:** 1 School of Medicine, Cardiff University, Cardiff, UK; 2 Department of Urology, University Hospital of Wales, Heath Park, Cardiff, CF14 4XW UK

**Keywords:** Urinary stone disease, Urolithiasis, Renal stones, Ureteric stones, Quality of life, Basic research, Patient-reported outcome measure

## Abstract

Urinary stone disease is a common, often recurrent disease, that can have a negative impact on patients’ health-related quality of life (HRQoL), often effecting working, productive members of society. The literature lacks data from structured, qualitative research which could give unique insight into patients’ HRQoL. The objective is to understand the impact of urinary stone disease and treatments on patients’ HRQoL, from patients’ and their relatives’ perspective using qualitative and quantitative methodologies. Semi-structured interviews and a focus group were used to understand the HRQoL issues of patients with urinary stones disease, covering the American Urology Association index stone categories. Thematic analysis was performed (using qualitative data analysis software). Familial impact was assessed using the family-related outcome measure (FROM-16©). 62 patients with stone disease and interventions (mean age 51, range 19–92) participated. Data collection stopped when data saturation was achieved. Analysis revealed negative impact of stone disease and interventions on the patients’ HRQoL, affecting domains of pain, physical symptoms, outlook on life, work/career, change in lifestyle/diet, social life, difficulties of daily living, travel/holiday problems, relationships and family member impact (106 themes grouped under ten broad headings). Sub-group analyses revealed similar impact in either sex, ureteric and renal stone groups. Recurrent stones were associated with work/financial concerns and treatment preferences varied accordingly. Our qualitative study presents detailed insights into the multidimensional impact of urinary calculi and their treatments on various domains of the HRQoL, confirming previous findings and adding new observations. The findings are expected to help in the development of patient-centric measures and communication tools.

## Introduction

The prevalence of urinary stone disease is high and is estimated to be 2–3% amongst the general population. The disease is a common cause for emergency hospital admissions with over 30,800 hospital admissions reported in England annually and 550,000 emergency room visits in the US in 2009 [1, 2]. The peak age of incidence is between 35 and 55 years. The recent evidence suggests that in western nations the incidence of renal stones has increased and women might be nearly as affected as men [3–5]. Risk of recurrence is estimated to be 50% within 5 years of the first stone episode. Given their age, patients with urinary stone disease are often working, productive members of society and if they require a leave of absence from employment, it could have economic ramifications on the individual, their family and on a wider, societal level [6].

Urinary stones present with a myriad of clinical features ranging from the pain of acute ureteric colic and systemic sepsis to minimal or asymptomatic disease. Once emergency treatment has been completed (treatment of infection, acute kidney injury and amelioration of pain), definitive treatment must be planned. This treatment pathway can be complex, multi-staged and can involve multiple hospital visits and admissions. Many patients suffer with recurrent/multiple stones requiring ongoing treatments. Stone disease and its treatment(s) can have an adverse effect on patients’ health-related quality of life (HRQoL) [7–12].

Various interventional treatments (shock wave lithotripsy and ureteroscopy) and non-interventional treatments exist, including expectant treatments. Patients with larger renal stones have the option of percutaneous nephrolithotomy (PNCL) and additional treatment options [13]. The treatment pathways’ success rates, as well as risks, are different with each treatment route.

The current pathway of care is suboptimal, considering the need to incorporate patient-reported outcomes, patient treatment preferences and resource efficiency. Most of the evidence is focused on the limited aspects of clinical management. In spite of clear indications from the literature for the need to understand patient perspectives in a scientific way on patients’ HRQoL (including the impact of disease and interventions), very few research groups have published peer-reviewed papers in this field [7–12]. This has made it difficult to evaluate and compare different treatments and technologies effectively. Many aspects of the treatments could be changed and traded if patient perspectives could be better understood. Currently, this results in the treatments being offered on an ad hoc basis, with patients being inadequately informed, while often putting them through protracted treatment journeys. There are also economic implications for possible inefficient use of resources.

Patient-reported outcome measures attempt to quantify patients’ health-related quality of life in a valid and reproducible way. Patients’ expectations of treatment and the impact of disease and their treatments on HRQoL are important outcome parameters. AUA guidelines on the treatment of urinary calculi state that treatment decisions should incorporate patient preferences to treatments, which would be influenced by quality of life [14]. Generic measures such as the SF-36 and the EQ-5D have been used to measure HRQoL but these measures tend to evaluate narrow aspects of the impact and often fail to elaborate on the clinically relevant domains. The impact of urinary calculi has been studied in more detail (using predominantly quantitative methods), in the recent years. These studies include Wisconsin Quality of Life (WISQOL), as well as questionnaires using the items from the PROMIS (Patient Reported Outcomes Information System) question bank in acute or chronic stages of stone disease [8, 11]. It has been suggested that the best treatment decisions are made when tailored to the needs and choices of individual patients [15, 16]. Qualitative research offers detailed understanding of patient views and is an essential first step in developing patient-centric measures. Qualitative research methods provide an opportunity for a systematic, in-depth evaluation of issues that may not be necessarily be answered through quantitative methods and are becoming increasingly common and valued in medical literature.

The aim of this study was to assess and understand the impact of urinary stone disease and treatments on patients’ quality of life from the perspective of patients and their family members. We expect the results to offer detailed insights into stone disease and form the foundations for the development of patient-centric measures and tools which will be used to facilitate good communication with patients about treatment options and ultimately improve patient care [7, 17–19].

## Methods

### Ethical considerations

Ethical and scientific approval was gained according to local guidelines (IRAS project ID 138478) for the conduct of qualitative study involving patient interviews and focus group discussion. Informed, written consent was gained and recorded. Data were handled in accordance to national data regulation guidelines.

### Participants

Patients suffering with urinary stone disease from the inpatient and outpatient settings of a university hospital stone management unit were invited to participate by a urologist with clinical responsibility for part or all of the participants care between and April 2014 and July 2016.

A list of inclusion and exclusion criteria is provided in Table 1. Patients were purposefully selected based on the nature of their disease to cover most of the index categories of stone disease. The groups sampled included those with renal or ureteric stones, symptomatic or asymptomatic disease with or without prior treatment. No payment was received for participation in this study, but travel expenses were offered. In addition, family members of 30 patients who were identified as next of kin, and attended with a patient, were invited to complete a family-reported outcome measure (FROM) questionnaire [20] in relation to their relative’s stone disease on a sequential basis, mostly in the clinic setting. The relative’s clinical status did not have a bearing on their inclusion/exclusion of the study.

**Table 1:** Inclusion and exclusion criteria

Inclusion criteria	Exclusion criteria
Adult patients (18 years upward)	Unable to give informed consent
Past or present urinary stone disease	Unable to speak English fluently
	Unable to give written consent

### Interviews and focus group

Patients participated in semi-structured interviews lasting between 30 and 90 min with a urology clinical research fellow trained in qualitative research methods. All interviews and the focus group took place in a quiet room in Urology outpatient department of the hospital or at patient’s houses. The interview schedule for the semi-structured interviews was created using the expert opinion of local urologists. Patients were encouraged to speak freely about acute and chronic health-related quality of life issues that they experience or have experienced due to their stone disease or its treatment. One focus group of eight participants was carried out as a consensus exercise.

### Qualitative thematic analysis

Interviews were digitally recorded, transcribed and thematic analysis was performed supported by qualitative data analysis software (NVivo 10 for Mac) in line with recommendations [21]. The stages of thematic analysis are seen in Table 2.

**Table 2:** Thematic analysis process [21]

1. Key themes or ‘big ideas’ identified following reading and re-reading the interview transcripts
2. ‘Units of information’ identified and highlighted (sentences/phrases) relevant to the research purposes
3. Selection of category headings to sort and group ‘units of information’
4. ‘Units of information’ coded into category headings to enable units to be placed within a category
5. Negotiation between research team members to the headings that most economically accommodate ‘units of information’
6. Categories generated in the first phase of data analysis are reviewed and revised

Transcripts were examined to identify key themes and coded by a single researcher. Interviews continued in each group, until saturation was reached and no new themes emerged, as is the current standard in qualitative data analysis in health settings [22–24]. Ten percent of interviews were analysed by a second coder to ensure consistency and quality assurance [25]. Key themes were tabulated and those mentioned by three or more patients were considered common. Common key themes were converted into questions and consolidated under broad headings. These broad headings were then grouped into overall domains.

### Family-related outcome measure (FROM-16©)

The FROM-16© questionnaire is a generic, validated tool used to assess the effect a patient’s disease has on a family member in two domains—‘emotional’ and ‘personal and social life’ [20]. FROM-16© was completed by a patient’s relative either in the clinic or inpatient setting.

## Results

Seventy-four patients were invited to participate. Sixty-two patients took part in interviews with a mean age of 51 (range 19–92). Ten patients declined, with reasons stated being ‘not interested’ and ‘lack of time’. Eight patients (five had taken part in the interviews, three had not) participated in a consensus focus group and no new themes were elicited during the process. Four patients were asymptomatic at the time of interview and 58 were symptomatic. Twenty-one participants were female (33%) and 41 male (67%). Twenty were first-time stone formers (32%) and 42 recurrent stone formers (68%). Three patients suffered with cystinuria (5%) and one patient had the diagnosis of distal renal tubular acidosis (2%).

### Themes

From the interviews, 106 themes were generated. These were grouped together under 10 broad headings (Table 3) and within three overall domains; ‘physical’, ‘psychosocial’ and ‘other’.

**Table 3:** Broad theme headings

Physical symptoms other than pain
Outlook on life
Pain
Work and career
Change in lifestyle or diet
Mental and emotional symptoms
Social life
Difficulties with activities of daily living
Travel or holiday problems
Relationships

Within the broad heading of ‘physical symptoms other than pain’ nausea and vomiting, frequency/urgency of urine and haematuria were the most commonly reported quality-of-life issues. Under the broad category of ‘pain’, acute and intense pain was the by far the most common hindrance to good quality of life with intermittent/chronic pain coming second. Within the ‘outlook on life’ theme, fear of the unknown and the fact that it seemed like ‘there was no end to the disease’ were the most common problems. With regard to ‘work’, the need to take time off work and the interference the disease had on career were most problematic. Loss of income is also a common problem encountered (although this was seen more in patients that were self-employed). Examples of quotes for different broad theme headings are seen in Table 4.

**Table 4:** Major themes, sub-themes and example quotes

Broad theme headings	Number of patients reported	Example quote
Physical symptoms: pain	56	“I was curled up in a ball saying I just needed some painkillers, some relief just to relieve it” Patient 007, male 61 single ureteric stone
Outlook on life	53	“It is like a ticking time bomb that is just hovering over you and you are just waiting for your next hospital admission…” Patient 065, female 39, recurrent stone former
Physical symptoms other than pain	51	“… all of a sudden I’ve had an urge to go to the toilet and before I’ve had a chance to turn around to walk towards the toilet, this just comes from me and it’s been really embarrassing” Patient 032, female 25, recurrent stone former “It’s like an irritation and if, you feel like you’re are bursting, but when you go, there’s hardly anything there” Patient 003, male 49, recurrent stone former
Work and career	35	“You are worrying about when the next one is going to come, how that is going to impact on your future, like I am trying to progress in work now, go up the ladder in work” Patient 065, female 39, recurrent stone former
Change in lifestyle or diet	46	“… my urine is always quite yellow, I am probably dehydrated quite a bit” Patient 061, male 30, recurrent stone former “I drink if I am thirsty you know if I am not thirsty I won’t just drink for the sake of drinking it, I don’t know maybe that’s the problem I’ve got maybe I should just drink anyway regardless but it’s hard for me to come and get a drink if I am not feeling thirsty” Patient 021, male 46, recurrent stone former
Mental and emotional symptoms	40	“I am worried like as soon as I start getting like a little pain in my stomach I am panicking that I am gonna have to go into hospital and I hate coming into hospital I like hate having to miss out on things my daughter does because I am stuck in hospital” Patient 032, female 25, recurrent stone former “… sometimes you are in bed and you can’t get out, but I make myself get out of bed and not think about what I am thinking and within 2 weeks it has gone!” Patient 074, male 33, recurrent stone former
Social life	31	“… if you go for a walk in the country, you can’t really do that because you are drinking water you have got to pee somewhere and that means you know there’s no toilets around, so it does restrict your activities” Patient 033, female 49, recurrent stone former “I feel lazy and tired and just isolated in a way and that is why I have kept myself away from so many people Hmm Which ain’t a good thing because they are my friends and I ain’t seeing them” Patient 036, male 34, recurrent stone former
Difficulties with activities of daily living	25	“When you’re having a really bad attack there is nothing much you can do except sit down and feel sorry for yourself” Patient 010, female 66, recurrent stone former “So even shopping, shopping, didn’t do shopping on my own… food shopping because of lifting heavy bags so unfortunately my husband had to come and help out!” Patient 017, female 54, non-recurrent stone former
Travel or holiday problems	19	“I didn’t want while I was going through treatment I didn’t want to have flare up while I was abroad and have to worry about you know picking up any type of infection or things we thought let’s get this sorted and then we can get back to how we were” Patient 029, female 50, recurrent stone former
Relationships	13	“Yeah financially we suffered, and it put a big strain on our relationship like as well because we just couldn’t afford to keep our head above water…” Patient 032, female 25, recurrent stone former

### Sub-group analysis

#### Ureteric vs renal stones

Patients with ureteric stones complained most about problems with work, particularly needing to take time off work. Pain and other physical symptoms were the second most problematic quality-of-life issues faced. Patients with renal stones at the time of the interview reported that physical symptoms other than pain were the worst HRQoL issue faced. Outlook on life was the second most reported issue, with fear of the unknown being the greatest problem under this heading. Pain was the third most common for patients with renal disease. However, it was important to note that both the groups had similar domains of QoL affected by the disease. Similarly, many patients, with recurrent disease reported to have experience of both ureteric and renal stones over a long period of time and the QoL issues appeared to be interlinked.

#### Age

For the youngest group of patients (younger than 35), physical symptoms other than pain were most commonly reported, followed by a negative outlook on life. Work was the third most important HRQoL issue in this age group with the inability to work, loss of income and the fear of losing their job being the worst aspects of this problem. For patients aged 36–60, physical symptoms other than pain and problems with work were most reported, with the need to take time off work and the fear of financial stress being major HRQoL difficulties. Pain was the third most important issue in this age group. Patients aged older than 61 felt the most difficult aspects of poor HRQoL were due to pain, other physical symptoms and a poor outlook on life.

#### Recurrent vs first-time stone formers

There was little difference reported between the HRQoL issues faced by recurrent and first-time stone formers. However, recurrent stone formers were much more concerned about the effect their stone disease had on their job/careers, with the major concerns being the need to take time of work and the financial instability associated with it.

#### Duration of disease

Patients were split into those suffering with urinary stone disease for less than a year, 1–3 years and more than 4 years. For those patients who had been suffering with their disease for less than 1 year and 1–3 years, no difference was seen in HRQoL problems. However, for those who had been suffering for the more than 4 year, the work and a negative outlook on life appeared to have a greater impact on the HRQoL of patients.

#### Patient communication and treatment preferences

Fifteen patients felt that they had not been given enough information before choosing treatment options in the past and six reported getting conflicting advice on treatment and stone prevention from different health professional (GPs, primary care nurses, emergency room doctors and urologists). Eleven patients reported getting information from the internet, 3 from their GP, and 15 from the hospital staff, 7 of whom reported receiving and reading hospital information sheets.

Patient preferences were varied. Nine patients preferred the least painful/invasive treatment and 16 felt they would like to avoid an operation/general anaesthetic. Sixteen felt they would simply follow the surgeon’s advice.

#### FROM survey results

Patient’s family members’ scores reflected that they were not overly affected by their family members’ disease. Participants reported a marginally greater degree of change in the ‘emotional’ domain compared to the ‘personal and social life’ domain. Participants scored questions regarding ‘being worried and sad’ highest and ‘difficulty with eating and travel’ lowest.

## Discussion

Our study demonstrates that stone disease and interventions tend to have significant impact on different domains of health-related quality of life. We found that there were few differences between sub-groups in terms of the quality-of-life issues faced. Pain along with physical symptoms other than pain seems to have the greatest impact on the HRQoL of patients and issues regarding work seem to be important to all sub-groups. Missing days from work and the possible financial instability that may ensue has been highlighted by patients throughout the study. This might be because stone patients tend to be of working age with economic dependents.

In terms of the groups ‘with’ or ‘without’ current stones, patients without current stones were less likely to suffer from, or have lower intensity of physical symptoms (as expected), but did have impact of the stones or interventions on psychosocial domains. Overall, we did not find major differences in the themes. This warrants more research in future, quantitative studies.

Other important HRQoL issues that we elicited were those pertaining to patients’ general outlook on life and mental and emotional symptoms. Patients reported the most difficult issues being the fact that there seemed to be no end in sight with their disease and the fear of the unknown that comes with having such a debilitating disease, where acute exacerbations can occur at any time in an unpredictable manner.

It is also important to note that in many, patients with urinary stone disease tend to suffer on a chronic basis. Patients probably learn multiple coping mechanisms related to their stone disease and this will impact on the quantification of their quality of life—they may score the same quality-of-life issue more favourably after a period of time as their expectations have been readjusted after living with the disease. Schwatrz et al. described this as ‘Response Shift Theory’ in 2007 [26]. With this in mind, it would be useful to undertake longitudinal studies to evaluate the longer term impact of urinary stone disease.

The data gathered from the FROM-16© questionnaire showed that urinary stone disease had little impact on family members in either domain. This could be accounted by the fact that patients tend to be most anxious before a diagnosis is made and the family members that were approached after initial diagnosis. Administering the FROM-16© questionnaire to patients in the emergency department could give a clearer picture of what effect the patient’s disease has on their relative(s) in the acute setting.

When considering the treatment of urinary stone disease, extra attention should be given with respect to patient education and quality of life. Patients with renal stones who are not experiencing an acute exacerbation may suffer slightly impaired quality of life (due to ‘niggling’ renal angle pain etc.), however, after treatment (ESWL, ureteroscopy), the HRQoL may deteriorate due to instrumentation/ureteric stone fragments. In this scenario, the HRQoL of the patient may be greatly impacted in the short term and may mimic the stone disease itself. Patients not warned of this prior to treatment may regret the decision to undergo any intervention in the first place. Good patient communication and pre-treatment counselling are the foundation of shared decision making and a studies has shown that good patient education may ameliorate ureteric stent-related symptom morbidity [27]. Patient education prior to urolithiasis interventions could similarly help as there is a wide variety of potential influences on patient choice.

Penniston et al. reported in 2012 that the most important factors impacting on quality of life of their stone population were patients’ concern about general health, pain, anxiety about current and future disease, decreased ability to focus on work, difficulty sleeping and lack of freedom to travel and attend social events. Many of these themes were confirmed to be similar to the ones identified in our population. However, in addition, we identified new findings related to the disease as well as the interventions and their complex interaction. Our study suggests that in our population sample, there were many other or related issues important to the patients. Similarly, we identified that the impact of current or past interventions, and indwelling stents can influence the intensity and the type of impact on different domains. We found that on many occasions, the impact tends to change during the treatment journey.

The most import HRQoL issues were pain, other physical symptoms, lack of support network, difficulty of disease prevention, difficulties with work and career and anxiety about the future. The item generation process of this study was patient-centred with open-ended questions employed as to give the most accurate and in-depth view of what patients felt were their most important HRQoL issues.

Our study has limitations. The items generated from the study were produced from English-speaking patient population in the United Kingdom. Patients from other countries and healthcare settings might have issues that were not picked up within our population. However, our response rate was excellent and therefore our data fairly represents the urinary stone formers within our population.

Difficulties were seen when attempting to disentangle HRQoL issues created from treatment from those associated with ureteric stent-related symptoms (a common adjunct used during the treatment of stone disease). All but the most veteran patients are unable to say which HRQoL issues were caused by ureteric stents and which were caused by the treatment itself. A possible solution to this problem would be the use of a stent-specific outcome measure alongside the proposed outcome measure when a stent (or stents) are deployed within the ureter [19].

Current methods of quantifying HRQoL in stone patients include the use of generic quality of life measures such as the SF-36 and EuroQol 5-Dimension (EQ-5D) [7]. New data have emerged from the disease-specific measures such as WISQOL and the use of PROMIS question banks. Many of the themes that were identified in our patient population have been identified as important QoL domains, as discussed earlier in the discussion. Future data from the quantitative and interventional studies would help to confirm these findings.

The findings from the present study provide insights into aspects of HRQOL issues associated with the urinary stone disease and different treatments. It should be viewed as part of a fundamental shift in research from typical limited outcome studies, to a more patient-centred approach. It has formed the framework for our work on the new universal ‘Urinary Stone and Intervention Quality of Life measure’ (USI-QoL) patient-reported outcome measure. Similarly, it offers the basis for the development of patient-centric measures such as patient-reported experience measures, decision aids and patient information tools.

## Conclusion

We have exhaustively elicited the most important HRQoL issues faced by patients with urinary stone disease in our population. These data could help to form the framework for patient-centric measures and counselling tools for patients with urinary stone disease.
